# High Throughput Random Mutagenesis and Single Molecule Real Time Sequencing of the Muscle Nicotinic Acetylcholine Receptor

**DOI:** 10.1371/journal.pone.0163129

**Published:** 2016-09-20

**Authors:** Paul J. Groot-Kormelink, Sandrine Ferrand, Nicholas Kelley, Anke Bill, Felix Freuler, Pierre-Eloi Imbert, Anthony Marelli, Nicole Gerwin, Lucia G. Sivilotti, Loren Miraglia, Anthony P. Orth, Edward J. Oakeley, Ulrich Schopfer, Sandra Siehler

**Affiliations:** 1 Musculoskeletal Disease Area, Novartis Institutes for BioMedical Research, Basel, Switzerland; 2 Center for Proteomic Chemistry, Novartis Institutes for BioMedical Research, Basel, Switzerland; 3 Analytical Sciences and Imaging, Novartis Institutes for BioMedical Research, Basel, Switzerland; 4 Center for Proteomic Chemistry, Novartis Institutes for BioMedical Research, Cambridge, Massachusetts, United States of America; 5 Genomics Institute of the Novartis Research Foundation, Novartis Institutes for BioMedical Research, San Diego, California, United States of America; 6 Department of Neuroscience, Physiology and Pharmacology, University College London, London, United Kingdom; Cinvestav-IPN, MEXICO

## Abstract

High throughput random mutagenesis is a powerful tool to identify which residues are important for the function of a protein, and gain insight into its structure-function relation. The human muscle nicotinic acetylcholine receptor was used to test whether this technique previously used for monomeric receptors can be applied to a pentameric ligand-gated ion channel. A mutant library for the α1 subunit of the channel was generated by error-prone PCR, and full length sequences of all 2816 mutants were retrieved using single molecule real time sequencing. Each α1 mutant was co-transfected with wildtype β1, δ, and ε subunits, and the channel function characterized by an ion flux assay. To test whether the strategy could map the structure-function relation of this receptor, we attempted to identify mutations that conferred resistance to competitive antagonists. Mutant hits were defined as receptors that responded to the nicotinic agonist epibatidine, but were not inhibited by either α-bungarotoxin or tubocurarine. Eight α1 subunit mutant hits were identified, six of which contained mutations at position Y233 or V275 in the transmembrane domain. Three single point mutations (Y233N, Y233H, and V275M) were studied further, and found to enhance the potencies of five channel agonists tested. This suggests that the mutations made the channel resistant to the antagonists, not by impairing antagonist binding, but rather by producing a gain-of-function phenotype, *e*.*g*. increased agonist sensitivity. Our data show that random high throughput mutagenesis is applicable to multimeric proteins to discover novel functional mutants, and outlines the benefits of using single molecule real time sequencing with regards to quality control of the mutant library as well as downstream mutant data interpretation.

## Introduction

Obtaining the detailed structure-function information that is needed in order to design ligands to target multimeric transmembrane proteins remains challenging. Structure determination of these proteins by X-ray crystallography or cryo-electron microscopy is a low-yield, slow technique. The identification of functionally important domains or residues in the protein is generally derived from site-directed mutagenesis followed by functional testing. This is also a labor-intensive process, and therefore it is common to see studies limited to small sets of mutations, often alanine scans, of regions hypothesized to be important. Carrying out unbiased, large scale mutagenesis studies clearly requires high throughput (HT) techniques to be practicable. We have previously applied HT mutagenesis by error-prone PCR to monomeric proteins, namely the G protein-coupled human prostacyclin receptor and the ion channel anoctamin 1 (ANO1) [1,2]. In both cases, we were able to identify residues important for receptor function and to derive structure-function relationships in the absence of crystal structures. Error-prone random PCR represents an HT-amenable approach to create large mutant libraries with coverage of all amino acids of a protein’s coding region. The aim of this study was to expand the application of the method to a multimeric protein, for which the pentameric human muscle nicotinic acetylcholine receptor (nAChR) was chosen.

The adult form of the muscle nAChR consists of four different subunits forming a pentamer around the channel pore, (α1)_2_β1δε, and each subunit contains four transmembrane stretches [3–5]. The nicotinic channel is permeable to cations, and is activated by acetylcholine at the neuromuscular junctions of skeletal muscle. The pentamer contains two orthosteric agonist binding sites in the N-terminal extracellular domain, at the interfaces of the α1-ε and α1-δ subunits [4,5]. We decided to use this well-characterized channel to test our HT random mutagenesis strategy and its associated functional screen, and chose to mutate the subunit that provides the principal side to both binding sites, *i*.*e*. the α1 subunit (457 amino acids). A library of 2816 mutant plasmids was generated and transiently transfected into cultured cells to allow us to perform an ion flux assay. Conditions for this assay were established using the wildtype α1 subunit co-transfected with a multigene-plasmid containing all three wildtype β1, δ, and ε subunits, and the nicotinic agonist epibatidine to activate the channel [6].

As a test of our HT mutagenesis strategy, we chose to search for mutations that conferred resistance to α-bungarotoxin (α-BTX) or tubocurarine, two orthosteric nAChR blockers whose binding determinants are well characterized [4,7,8]. α-BTX is a snake venom protein that binds irreversibly to muscle nAChR. Residues important for its binding to the extracellular domain of the mouse α1 subunit have been confirmed by the co-crystallization data by Dellisanti *et al*. [9]. The small alkaloid ligand D-tubocurarine binds reversibly. Again, several α1 subunit amino acids have been identified as important for tubocurarine binding on the basis of functional assays and confirmed by the crystal structure of tubocurarine bound to the ACh binding protein, a snail soluble protein orthologous to the extracellular domain of nAChRs [8,10,11]. α1 subunit mutants that still responded to the agonist, but had decreased sensitivity to either inhibitor were identified in the screen, confirmed and further characterized.

**Fig 1:**
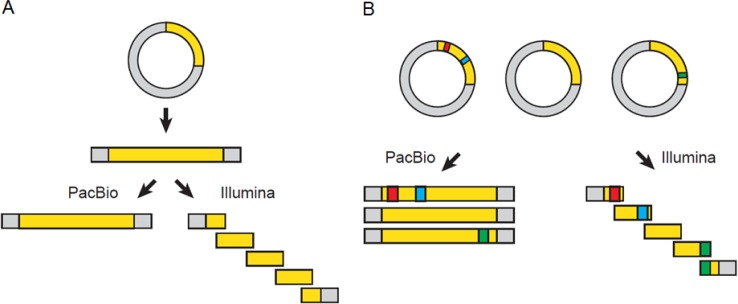
Principles of SMRT sequencing and other NGS methods. Schematic drawing of SMRT sequencing (PacBio RSII) and other NGS techniques such as an Illumina 454 reader. (A) The complete region covering the full-length coding sequence of the α1 subunit in each mutant cDNA (1371 bp) was sequenced using SMRT sequencing, whereas only up to 300 bp stretches would have been read by an Illumina reader. (B) SMRT sequencing can link each of three fictive mutations (red, blue, green) to the individual mutant, whereas this information is lost with Illumina sequencing due to the amplification of short stretches only.

The novelty of the present study lies not only in the application of HT mutagenesis to a multimeric protein complex, but also in the first application of single molecule real time sequencing (SMRT) to sequence all the 2816 α1 subunit mutants. Mutant libraries had been previously characterized using ‘Next Generation Sequencing’ (NGS) techniques, such as an Illumina 454 reader [1,2]. Illumina sequencing is based on the amplification of the cDNA molecules with an engineered DNA polymerase and can read stretches of 50–300 base pairs (bp). This implies that a typical membrane protein such as the nAChR α1 subunit (1371 bp) needs to be sequenced in multiple parallel reactions, and this makes it impossible to distinguish single plasmids within the pooled mutant library (Fig 1). On the other hand, the novel SMRT sequencing using a PacBio RSII reader can read individual DNA molecules up to 40’000 bp sequences in a single reaction, thereby preserving the information of the origin of each mutation [12,13]. In the study described here, SMRT sequencing allowed us to see which mutations occurred together on each single α1 subunit mutant cDNA plasmid, and hence to deconvolute data from pooled plasmid sequencing.

## Material and Methods

### Cloning of wildtype subunit plasmids

The cDNAs for the four human muscle nAChR subunits α1 (CHRNA1, NM_000079.3), β1 (CHRNB1, NM_000747.2), δ (CHRND, NM_000751.2), and ε (CHRNE, NM_000080.3) were synthesized by GENEWIZ (Europe), and a Kozak consensus sequence (GCCACC) was added upstream of each start codon. cDNA sequences were cloned into cytomegalovirus promotor (CMV)-driven single expression vectors. A triple multigene expression vector containing β1, δ, and ε subunits, each linked to an elongation factor (EF)-1α promotor was engineered for combination with a single CMV-dependent α1 subunit vector. Finally, a quadruple gene vector containing the CMV-α1 expression cassette upstream of the EF1α-β1, EF1α-δ, and EF1α-ε expression cassettes was created as a control (S1A Fig). All plasmid inserts were sequence-verified. Amino acid (AA) and nucleotide (nt) numbering for the α1 subunit in this study is based on counting the starting methionine or the first nucleotide of the start codon ATG as ‘1’. The full-length human amino acid sequence used in this study has been aligned to the mouse equivalent, and can be found in the supplement (S2 Fig).

### Design of α1 subunit mutant library

The mutant library for the human α1 subunit was generated at Solvias AG (Switzerland) using error-prone PCR based on the Genemorph II random mutagenesis kit and 500–1500 ng plasmid DNA. PCR products were purified with a kit (Qiagen), and ligated into the parental CMV-containing vector at an equivalent position. Mutant DNA plasmids were transformed into *Escherichia coli*. 48 mutants were sequenced to calculate the mutation rate for this subset using the HT Mutagenesis Simulation Program (HT-MSP) as described previously [1,2]. The library with the desired mutation rate (made from 1500 ng DNA) was up-scaled to obtain 2816 α1 subunit mutants in a 96 well format as previously described [1]. Glycerol stocks were frozen to secure the library. The cDNA concentrations of all mutants were normalized to 40 ng/ μl. Finally, 1 μl aliquots were taken from each mutant in order to create four pools (each containing 704 mutants), cDNAs were linearized, and subjected to SMRT sequencing.

### ‘Single molecule real time’ sequencing and data analysis

SMRT sequencing was performed based on the manufacturer’s protocol. High quality consensus reads were extracted from the raw sequence signals using the SMRT analysis 2.3 patch 4 on DNAnexus (Pacific Biosciences, USA). Reads were required to have four full passes and 90% consistency in base calls, ensuring high quality initial reads. Single-molecule reads were aligned to the wildtype α1 subunit sequence in order to identify mismatches in all reads. Examining the total number of observed events for each possible specific mutation produced a bimodal distribution of sequencing errors and true mutations. A new custom clustering algorithm was developed in order to control shifting of reads between clusters, and hence to overcome the overlap of true mutations and sequencing noise (1–5 errors per molecule). For this, reads farthest from cluster medoid were placed into a background noise bin cluster, which allowed the creation of new and destruction of existing clusters. The likelihood of observing the reads was calculated over the foreground and background clusters, and sequences were reassigned until convergence. Therefore, prior probability for observed reads per cluster was specified, and terms for the inferred number of clusters without mutations and total reads falling into them included. The resulting set of clusters represented 1929 sequences with at least one mutation and 887 without, matching the expected Poisson distribution shape.

### Cell culture and transfection

Parental human embryonic kidney (HEK)293 cells were obtained from American Type Culture Collection (ATCC; CRL-1573), and cultured in Dulbecco’s modified Eagle medium (DMEM) supplemented with 10% (v/v) fetal calf serum (FCS) at 37°C in a 5% CO_2_/ 95% humidity atmosphere. For passaging, cells were detached from the cell culture flask by washing with phosphate-buffered saline (PBS) without Ca^2+^/Mg^2+^, and brief incubation with 0.25% trypsin/ 1 mM EDTA. All solutions were from Life Technologies with the exception of FCS (Bioconcept). For assay plates, cells were seeded into poly-D-lysine coated 384 well plates at 6000 cells/well in 40 μl culture media using an automated cell culture system (SelecT). 24 h later, cells were transiently transfected using human muscle nAChR cDNA plasmids and Lipofectamine-LTX (Invitrogen) using a recommended transfection protocol adapted to a 384 well format. Cells were subjected to the ion flux assay or immunostaining 48 h post-transfection.

### Ion flux assay

A Ca^2+^ flux assay for the human muscle nAChR channel was developed using an assay buffer depleted of Na^+^ and the Ca^2+^-specific fluorogenic dye Fluo-4. Cells in the 384 wells were washed twice with 80 μl assay buffer (20 mM HEPES, 0.9 mM KH_2_PO_4_, 0.8 mM MgSO_4_, 3 mM CaCl_2_, 25 mM glucose, 4.5 mM KCl, 130 mM N-methyl-D-glucamine, pH to 7.4) using an automated plate washer (Tecan), and leaving 20 μl residual buffer in the wells. 20 μl of assay buffer containing 3.2 μM Fluo-4 acetomethylester and 2.5 mM probenecid were added, and cells incubated for 1 h at 37°C, 5% CO_2_, 95% humidity. Cells were washed with the previous procedure and 20 μl residual buffer, and 15 min later the agonist epibatidine (or other) diluted in assay buffer was injected in a 10 μl volume on a fluorescence imaging plate reader (FLIPR; Molecular Devices). Inhibitors were added 30 min before applying an EC_80_ concentration of epibatidine (determined by a separate experiment at the beginning of each experimental day, and ranging from 100–270 nM), and in the case of α-BTX 0.1% BSA was added. α-BTX was purchased from R&D System, (+/-)-epibatidine and D-tubocurarine from Anawa, and anatoxin, cytisine, and dimethylphenylpiperazinium (DMPP) from Tocris. The intracellular de-esterified Ca^2+^ dye was excited at 488 nm. Baseline fluorescence signals (F_b_) were recorded in 2 sec intervals for 10 sec using a band spectrum filter (510–570 nm), and fluorescence signals after agonist addition for 60 sec in one sec intervals in order to determine the maximal fluorescence (F_max_). The FLIPR 2.2.3 software was used to visualize Ca^2+^ flux kinetics, and to extract F_b_ and F_max_ in order to calculate Ca^2+^ signals normalized to the resting Ca^2+^ level as (F_max_—F_b_)/ F_b_. Concentration response curves (n = 3–5 per experiment) were analyzed by non-linear regression curve fitting using the GraphPad Prism 6.04 software.

The library was split into sets of 88 mutants, each of which was expressed four times in the four quadrants of a 384 well plate, allowing for testing of the same set of mutants in four different conditions per plate. For the mutant screen, each plate quadrant corresponded to one condition: (1) buffer control, (2) epibatidine EC_80_ control, (3) epibatidine EC_80_ + 0.1 μM α-BTX, (4) epibatidine EC_80_ + 50 μM tubocurarine. Each assay plate contained also 32 control wells, the latter expressing wildtype muscle nAChR, and eight wells thereof subjected to each of the four conditions as outlined above. Mutant hits were selected if they had a detectable epibatidine response, and impaired inhibition of epibatidine-stimulated Ca^2+^ signals in the presence of either α-BTX or tubocurarine compared with the wildtype channel control.

### Immunostaining of the α1 subunit of muscle nAChR in transfected cells

Cells were fixed by adding PBS containing 4% (v/ v) paraformaldehyde and incubating at room temperature (RT) for 20 min. Cells were washed three times with 50 μl PBS before blocking with 10% FCS and 0.2% Triton X-100 in PBS for 30 min at RT. The blocking solution was discarded before addition of an anti-α1 subunit-specific monoclonal rat antibody (1:600; clone 210; Covance) in blocking buffer for 2.5 h at RT (the antibody recognizes the residues 371–386 of the α1 subunit). Cells were washed before incubation with an AlexaFluor488-conjugated goat-anti–rat antibody (1:500; Life Technologies) in PBS containing 1% FCS and 0.02% Triton X-100 for 1 hour at RT. Cells were washed, 50 μl PBS was added, and immunostaining microscopy was performed using an Axiovert 25CFL microscope (Zeiss). Images were collected with a LSM 510 laser system at a 40x magnification using excitation at 488 nm and measuring emission at 530 nm.

## Results

### Development of a functional HT assay for transiently transfected muscle nAChRs

A Ca^2+^ flux assay suitable for HT mutant screening was established using an assay buffer depleted of Na^+^, the nAChR-selective agonist epibatidine, and HEK293 cells. Co-transfection of single plasmids for the four subunits of the muscle nAChR gave functional responses that were too small for robust screening (S1B Fig), despite extensive attempts at optimizing transfection and assay conditions (data not shown). Cloning of all four genes into the same plasmid, however, improved Ca^2+^ signals by about three-fold. In order to enable screening of α1 subunit mutants in a separate expression vector, an additional triple subunit plasmid for wildtype β1, δ, and ε subunits was constructed. Co-transfection of the latter together with a single wildtype α1 subunit plasmid yielded epibatidine-stimulated Ca^2+^ responses that were as high as seen with the quadruple gene plasmid. As expected, no significant signals were seen when transfecting the triple β1-δ-ε plasmid in the absence of the α1 subunit. Optimal assay conditions included seeding of 6000 cells in each of the 384 wells one day before transfection. For transfection 50 ng cDNA per well (DNA:lipofectamine ratio of 1:3.5) were used, and the functional assay was performed two days post-transfection.

The pharmacology of the muscle nAChR was characterized in the transient Ca^2+^ flux assay, and an EC_50_ of 90 nM (± 3.8, n = 5) for the agonist epibatidine was determined (S1C Fig). For characterization of the two inhibitors, an EC_80_ concentration of epibatidine was used, which was determined from a concentration response curve on each experimental day. For the wildtype receptor, the IC_50_ values of α-BTX and tubocurarine were found to be 2.9 nM (± 0.79, n = 3) and 3.2 μM (± 2.4, 3 n = 3), respectively (S1D Fig), and concentrations of 0.1 μM α-BTX and of 50 μM tubocurarine were utilized in the mutant screen. The potency of epibatidine and α-BTX are broadly consistent with literature data (EC_50_ of 260 nM and IC_50_ of 5 nM, respectively) obtained by a similar technique in TE671 cells that endogenously express the human fetal muscle nAChR type containing a γ instead of an ε subunit [14]. For tubocurarine, IC_50_’s described for electrophysiology assays for the mouse adult muscle nAChR are 40–50 nM [15,16], and differences might be species- and/or assay-related.

### Generation of a muscle nAChR α1 subunit mutant library

The quality and coverage of a mutant library is defined by its size, coverage, mutation rate and bias. The final library should preferably contain at least one mutant for each amino acid (‘total coverage’), and ideally this mutation should occur as a single amino acid mutation, in the absence of any other mutations in the same plasmid (‘unique coverage’). Small libraries require high mutation rates to achieve optimal total coverage, but this often results in the accumulation of mutations, a drop in unique coverage and an increase in the risk of obtaining non-functional proteins. Low mutation rates give optimal unique coverage, but result in a high percentage of constructs without mutations, and therefore require large library sizes that are more challenging to screen. Given the maximal desired size of the library and the length of the protein or region of interest, the optimal parameters can be calculated using the HT-MSP program as described previously [1].

**Fig 2:**
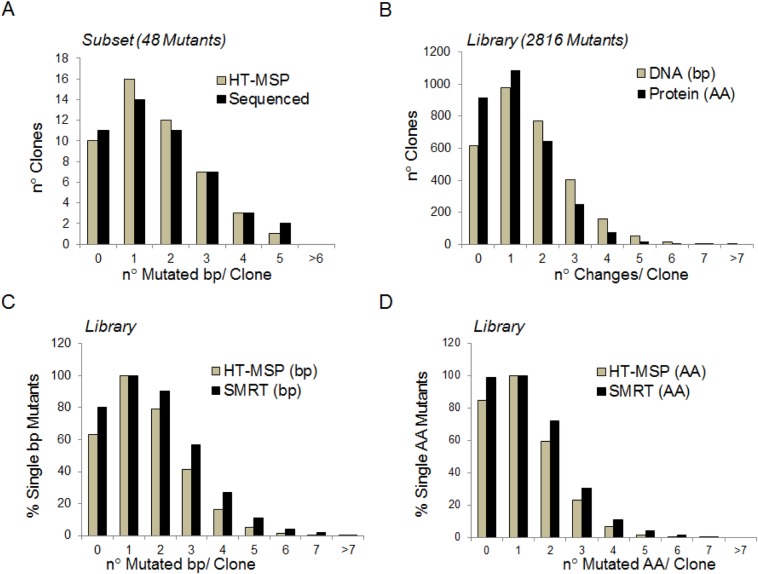
Error-prone PCR mediated mutagenesis of the nAChR α1 subunit. (A) Number and distribution of mutations per clone after error-prone PCR mediated mutagenesis of the nAChR α1 subunit. Black bars represent the number of mutations after standard full-length Sanger sequencing of 48 mutants. Grey bars represent the sequenced mutational spectrum at a mutation rate of 1.15/kb using HT-MSP [1]. (B) Expected number and distribution of nucleotide (grey) or amino acid (black) changes in the whole library predicted by HT-MSP with a mutation rate of 1.15/kb. (C, D) Comparison of the distribution of nucleotide (C) and amino acid (D) changes as predicted by HT-MSP (grey) and as measured by SMRT-sequencing (black) for the total library.

Error-prone PCR was used to generate a library of 2816 mutants for the 1371 bp-long α1 subunit coding sequence. Initial sequencing of a small subset of 48 mutants estimated the mutation rate to be 1.15 mutations per kb. Given the size of our library, this mutation rate would lead us to expect a unique coverage of 2.4 for the 457 amino acids in the α1 protein (*e*.*g*. each position would be found to be mutated 2.4 times on average), and a total coverage for each amino acid of 7.8. These estimates were found to be close to the actual measurements obtained for the full library by SMRT sequencing (mutation rate of 1.27/kb, unique and total coverages for each amino acid of 3.4 and 7.9, respectively). This increases our confidence in using the HT-MSP program to optimize the ideal mutation rate from a small subset of mutants before preparing a large mutant library (Fig 2). Furthermore, SMRT sequencing for the first time enabled the characterization of all individual mutants of a large mutant library by its ability to sequence the full-length coding region of each plasmid in one reaction. Therefore, using SMRT sequencing, multiple mutations within an individual plasmid can be detected, whereas other NGS techniques can yield only the overall types of mutations within a library.

**Fig 3:**
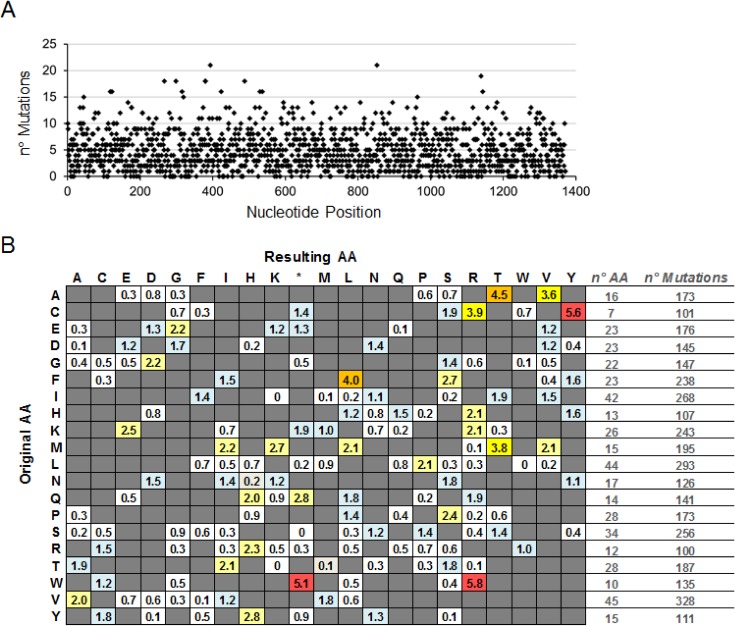
Mutational bias analyses of the α1 subunit mutant library. (A) Number of mutations for each of the 1371 nucleotides of the α1 subunit sequence according to SMRT sequencing data. (B) Frequency of amino acid (AA) changes found in all 2816 α1 subunit mutants. The left column shows the wildtype amino acid, whereas the top letters show the mutated amino acid with (*) representing a mutation into a stop codon. The absolute number (n°) of each type of amino acid within the α1 protein, and the absolute number of mutations for this amino acid are depicted on the right.

Detailed analysis of the mutant library sequences obtained by SMRT revealed that the distribution of mutations had minimal bias as expected for error-prone PCR [17], with a slightly higher abundance of transitions as compared to transversions (ratio 1.5), and a slight bias for the mutation of purines (A, T) over pyrimidines (G, C) (Table 1). At the nucleotide level 1269 or 93% of the 1371 nucleotides have been mutated between 1–21 times (Fig 3), resulting in a complete coverage of the 457 amino acids (*i*.*e*. each amino acid was mutated at least once) (S3 Fig). 139 of the 143 possible types of amino acid mutations that can be achieved by one nucleotide change per codon were found in the α1 subunit mutant library. This limitation in possible codon changes results in a bias in the type of mutation that can be achieved for each amino acid. Furthermore, the mutation frequency of each amino acid is determined by the number of possibilities of codon changes that result in non-homologous amino acid changes, as well as the mutational bias at the nucleotide level. The absolute number of mutations detected for each amino acid type was between 100 and 328. The mutation frequency for each mutation type is presented as the total number of mutations normalized to the absolute frequency of the amino acid in the sequence. The highest mutation frequencies of 5.8% and 5.6% were found for the tryptophan-to-arginine and cysteine-to-tyrosine mutations, respectively.

**Table 1:** Nucleotide mutation properties of the α1 subunit mutant library based on SMRT sequencing.

*Transition (Ts)*	*%*	*Transversion (Tv)*	*%*
A → G	54	A → T	39
T → C	56	T → A	38
G → A	70	A → C	7.3
C → T	63	T → G	6.1
		G → C	10
***Bias Indicator***		C → G	9.5
Ts/Tv	1.5	G → T	20
(AT → GC) / (GC → AT)	0.8	C → A	28
A → N, T → N	53		
G → N, C → N	47	***Mutation Rate***	1.27/kb

### Functional screen of the muscle nAChR α1 subunit mutant library

**Fig 4:**
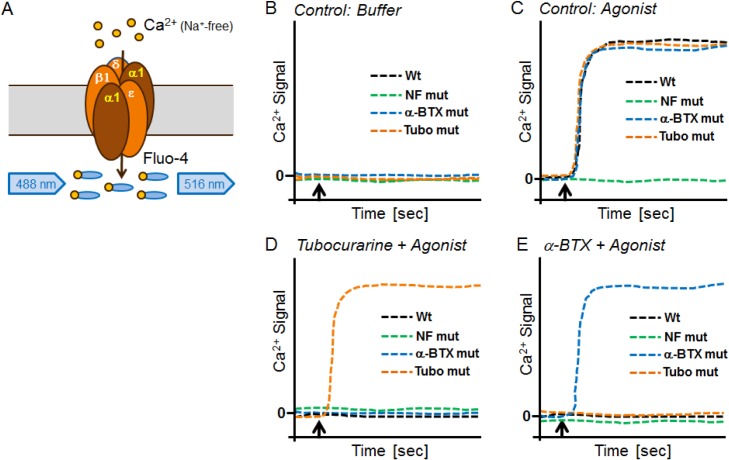
Illustration of the functional α1 mutant screen. (A) The muscle nAChR contains two α1 subunits that form a pentamer together with co-transfected β1δε subunits. Ca^2+^ flux through channels containing wildtype or mutant α1 subunits was measured in a Na^+^-free buffer using the Ca^2+^-dye Fluo4. Typical raw Ca^2+^ signal traces over time are presented for (B) the buffer control, (C) the agonist (epibatidine) control, (D) the blocker tubocurarine, or (E) the blocker α-BTX (both added 30 min before adding the agonist, the injection of which is indicated by an arrow). Agonist-stimulated Ca^2+^ signals of wildtype (Wt) muscle nAChR (black lines) are inhibited by tubocurarine or α-BTX. A non-functional mutant (green lines) is inactive in all conditions, and does not show any Ca^2+^ signals. An α-BTX resistant mutant (blue lines) or tubocurarine resistant mutant (red lines) shows agonist signals in the absence and presence of the respective blocker.

The 2816 α1 subunit mutants were transiently expressed together with the β1δε wildtype subunits of the muscle nAChR, and the channels tested for sensitivity to the two selected competitive blockers α-BTX and tubocurarine, in order to identify amino acids involved in binding of the antagonists. Mutant hits were identified on the basis of raw kinetic Ca^2+^ traces as schematically shown in Fig 4. Epibatidine-responsive mutants were eventually selected as hits if they were completely or in part resistant to blockade by the antagonists α-BTX and/or tubocurarine. Mutants were considered as hits, only if they responded to the agonist (epibatidine) in the control wells, which indicated that the mutant channel was functional and reached the cell surface. Around 40% of the mutants did not display a measurable agonist response. In other HT mutagenesis projects, such as ANO1, a similar percentage was observed, and immunostaining revealed that these mutant receptors were not transported to the cell surface [2]. Moreover, buffer control wells for all mutants were inspected to make sure mutants were not spontaneously active. Following the first screening round, bacterial glycerol stocks of preliminary mutant hits were used to prepare new cDNA plasmids for confirmation testing. Eight α1 subunit mutant hits were confirmed and sequenced and these consisted of six unique mutants for tubocurarine, one unique mutant for α-BTX, and one dual mutant (Table 2).

**Table 2:** Mutant hits identified from testing 2816 α1 subunit mutants in the functional screen.

Mutant	Mutated AA *(nt)*						
Mutant-1		**V197A** *(T590C)*			**V275M** *(G823A)*		
Mutant-2	**D44N** *(G130A)*	silent *(C402T)*				**L278Q** *(T833A)*	
Mutant-3			**Y233H** *(T697C)*				silent *(T1083A)*
Mutant-4		silent *(C603A)*	**Y233H** *(T697C)*				
Mutant-5		silent *(T333C)*			**V275M** *(G823A)*		
Mutant-6					**V275G** *(T824G)*	**S308F** *(C923T)*	**L453S** *(T1358C)*
Mutant-7				**I235M** *(C705G)*			
Mutant-8			**Y233N** *(T697A)*				

All mutant hits except mutant-7 were found in the tubocurarine screen. Mutant-6 and mutant-7 were identified in the α-BTX screen. Amino acid (AA) changes are shown in bold. Nucleotide (nt) alterations are indicated in italics, and if not leading to an amino acid change (‘silent’) are shown in grey.

### Analysis of the functional muscle nAChR α1 mutants

From Table 2 it is clear that Y233 and V275 are the main amino acid positions that were identified in our functional HT mutagenesis screen. In order to gain insight into the quality of our approach, we performed a full analysis of the SMRT data from our library to identify all clones carrying mutations in any of the amino acid positions identified in the eight hits (Table 3 and S1–S3 Tables). With regards to the V275 position (Table 3), our hits contained valine-to-methionine or valine-to-glycine mutations. In the analysis we found two more V275 mutants that were not picked up by the screen (SMRT-9 and SMRT-10). In these two clones, V275 was mutated to alanine or to leucine and additional mutations were present. We do not know whether the lack of detection of these two clones was due to the milder nature of the mutations at position 275 (as it was changed to a different amino acid), or to a compensating or dominant negative effect of the associated mutations. A similar analysis of position Y233 (S1 Table) gives a similar result: SMRT-20 (identical to Mutant-3) was missed in the screen, SMRT-22 was non-functional (Y233 is mutated into a stop codon), and SMRT-21 has additional mutations that could have rendered this mutant non-functional.

**Table 3:** SMRT analysis of all α1-V275 mutants in the library.

Mutant	Mutated AA *(nt)*										*n°* Reads
**Mutant-1**			**V197A** *(T590C)*		**V275M** *(G823A)*						10
**Mutant-5**	silent *(T333C)*				**V275M** *(G823A)*						12
**Mutant-6**					**V275G** *(T824G)*		**S308F** *(C923T)*		**L453S** *(T1358C)*		11
SMRT-1	**V111I** *(A306G)*	silent *(G331A)*	**V197A** *(T590C)*								14
SMRT-2	silent *(C321T)*	silent *(C369T)*	**V197E** *(T590A)*								20
SMRT-3	W169* *(G506A)*		**V197E** *(T590A)*								7
SMRT-4			**V197E** *(T590A)*	**K262E** *(A784G)*		**L293S** *(T878C)*					21
SMRT-5	**W138R** *(T412C)*		**V197E** *(T590A)*								15
SMRT-6			**V197E** *(T590A)*								22
SMRT-7		silent *(T465C)*	**V197E** *(T590A)*								7
SMRT-8			**V197M** *(G589A)*	silent *(G873A)*							10
SMRT-9	silent *(G210A)*	silent *(T498C)*		**M227T** *(T680C)*	**V275A** *(T824C)*	**S288P** *(T862C)*		**T348A** *(A1042G)*		**K360R** *(A1079G)*	13
SMRT-10				**C212G** *(T634G)*	**V275L** (G823C)						8
SMRT-11	silent *(A267C)*	**F155S** *(T464C)*					**S308P** *(T922C)*	silent *(G996A)*			12
SMRT-12	**N84D** *(A250G)*						**S308P** *(T922C)*				2
SMRT-13	**K145E** *(A433G)*					**F300V** *(T898G)*	**S308T** *(T922A)*	silent *(C1014T)*		**M349K** *(T1046A)*	8
SMRT-14				silent *(C738A)*			**S308Y** *(C923A)*			**M424V** *(A1270G)*	15
SMRT-15	**P89S** (*C265T)*	silent *(C654T)*		**T257I** *(C770T)*			**S308T** *(T922A)*	**H326N** *(C976A)*			16
SMRT-16	**H23L** *(A68T)*			**S246P** *(T736C)*					**L453S** *(T1358C)*		15
SMRT-17	**I100F** *(A298T)*	**T168A** *(A502G)*	silent *(C723A)*	**V281E** *(T842A)*		**F300S** *(T899C)*			**L453S** *(T1358C)*	**N454K** *(T1362A)*	7
SMRT-18		**Y132C** *(A395G)*		silent *(G690A)*		**V332A** *(T995C)*		**K400E** *(A1198G)*	**L453I** *(T1357A)*		13
SMRT-18	silent *(G147A)*	**N88K** *(T264A)*		**K96I** *(A287T)*					**L453I** *(T1357A)*		11

The 21 mutants include five α1-V275 mutants including mutant-1, mutant-5, and mutant-6, all hits discovered in the toxin screen. The library contained eight mutants for α1-V197 (including three single mutants in this position) and five and four α1-S308 and α1-L453 mutants, respectively. The 18 ‘SMRT’ mutants present in the library based on SMRT sequencing were not hits in the functional screen. Nucleotide alterations are indicated in italics, and silent amino acid changes in grey. The number (n°) of SMRT reads reflects the confidence of sequence determinations. (*) indicates a stop codon.

Interestingly, none of the mutants identified contained mutations at the residues known to interact with α-BTX or tubocurarine [4]. SMRT sequencing allowed to retrieve systematically from the library all the mutants at the 15 positions reported by Dellisanti *et al*. [9] to be involved in the binding of α-BTX to the monomeric extracellular domain of the mouse α1 subunit (S2 Fig). We found 130 mutants (24 of which are single amino acid mutants). None of these was a hit in the functional screen (Table 4). We cannot link the identified mutants to functional data. A lack of sensitivity of the functional screen could not explain why all these mutants were not found. A possibility is that these mutations reduced or abolished epibatidine sensitivity, and thus any effect on antagonist binding could not be detected.

**Table 4:** SMRT sequencing summary of α1 subunit mutants for published α-BTX binding site residues.

*AA*	*n°*	*Mutation (n°)*	*n° Single AA Mutant*
V111	4	V111I (4)	**0**
Y113	7	Y113N (2), Y113H (2), Y113S (1), Y113C (1), Y113* (1)	**1** (Y113H)
D119	6	D119E (3), D119V (2), D119G (1)	**1** (D119E)
F120	11	F120S (5), F120L (4), F120C (1), F120I (1)	**3** (2xF120L, F120S)
T168	11	T168S (4), T168A (3), T168I (2), T168N (2)	**3** (T168S, T168I, T168S)
W169	15	W169R (5), W169G (1), W169C (1), W169* (8)	**2** (W169R, W169G)
S207	12	S207T (4), S207P (4), S207F (3), S207A (1)	**3** (2xS207P, S207T)
V208	9	V208A (3), V208E (2), V208M (2), V208L (1), V208G (1)	**2** (V208M, V208A)
T209	10	T209S (5), T209A (2), T209I (2), T209P (1)	**1** (T209S)
Y210	8	Y210H (5), Y210F (1), Y210* (2)	**2** (Y210H, Y210F)
S211	5	S211T (3), S211F (1), S211P	**1** (S211T)
C212	13	C212Y (4), C212S (3), C212R (3), C212G (2), C212* (1)	**3** (C212S, C212R, C212Y)
P214	8	P214L (4), P214S (2), P214H (1), P214T (1)	**2** (P214S, P214L)
P217	4	P217L (1), P217S (1), P217H (1), P217T (1)	**0**
Y218	7	Y218N (4), Y218S (2), Y218H (1)	**0**
*Sum*	*130*	* *	*24*

Residues are corresponding to the 15 extracellular residues of the mouse α1 subunit described to interact with α-BTX [9]; numbering converted to the human sequence used here. The 130 mutants present in the α1 subunit library were identified based on SMRT sequencing. (*) indicates a stop codon.

Remarkably, all the positions identified are in the transmembrane domains: Y233 and I235 in M1, and V275 and V278 in M2. None of these residues are in the orthosteric binding site, and it is clear that none of these residues can physically interact with tubocurarine or α-BTX (S2 Fig). This begs the question of how they can reduce the channel sensitivity to these antagonists. Mutations here have been found to produce ‘Gain-of-Function’ (GoF) phenotype in (mouse) muscle nAChR α1 (V275C) [18]. To test if the functional screen indeed identified GoF mutants, the three most promising single amino acid mutants (Y233H, Y233H, V275M) from the screen were chosen, and tested for a GoF phenotype.

### Functional characterization of muscle nAChR α1 mutants

**Fig 5:**
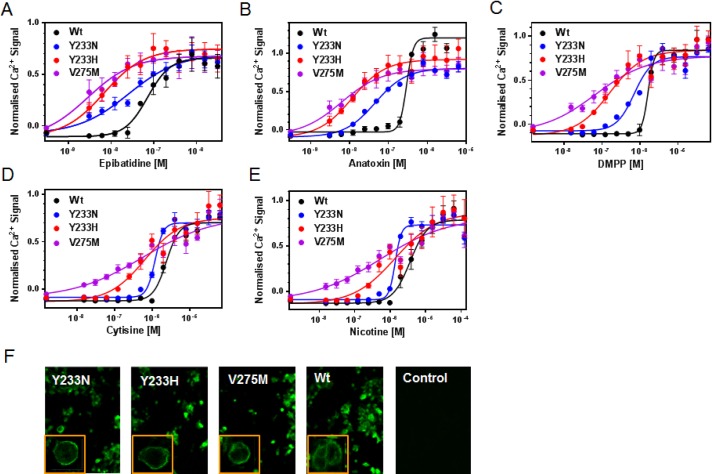
α1-Y233 and α1-Y275 mutants enhance potencies of channel agonists. (A-E) Concentration response curves of five agonists measured in the Ca^2+^ flux assay for transiently expressed wildtype and mutant α1-containing nAChRs. Basal fluorescence of each well was subtracted from maximal signals, and used to normalize signals from each well. All curves represent averages (± s.d.) of three independent experiments, and each experiment was conducted in triplicates. (F) Cells from the same transient transfections as used for the Ca^2+^ flux assay were immuno-stained and confocal pictures taken at 40x magnifications with inlets depicting one enlarged single cell.

Concentration response curves for five known muscle nAChR agonists were determined in the transient transfection Ca^2+^ assay for wildtype nAChR as well as the three selected α1-mutants. As can be seen in Fig 5 and Table 5, an increase in the potency of all five agonists was observed for all three mutants. For the most potent agonists, epibatidine, anatoxin and DMPP, the increase in potency was much greater with the α1-V275M mutation (17-33-fold shift) than with the Y233H (10-17-fold) or Y233N (2.9–4.4-fold) mutations. For cytisine and nicotine, the least potent agonists, differences between the mutants were less pronounced. α1 subunit-specific immunostaining indicated that all three mutants were expressed at the cell surface at levels similar to those achieved by wildtype α1 nAChRs. This suggests that the functional mutagenesis screen indeed identified GoF mutants.

**Table 5:** α1-Y233 and α1-Y275 mutants enhance the potency of nicotinic receptor agonists.

	α1-Wt	α1-Y233N		α1-Y233H		α1-V275M	
*Agonist*	*EC*_*50*_ *[nM]*	*EC*_*50*_ *[nM]*	**Fold↑**	*EC*_*50*_ *[nM]*	**Fold↑**	*EC*_*50*_ *[nM]*	**Fold↑**
Epibatidine	*72 ± 39*	*27 ± 11*	**2.9** ± 1.0	*7 ± 0*	**10** ± 0.4	*4 ± 1*	**17** ± 4.7
Anatoxin	*191 ± 59*	*46 ± 1*	**4.4** ± 0.1	*12 ± 1*	**17** ± 0.7	*6 ± 2*	**33** ± 9.7
DMPP	*1630 ± 203*	*705 ± 22*	**2.3** ± 0.1	*161 ± 10*	**10** ± 0.6	*67 ± 15*	**25** ± 5.2
Cytisine	*2350 ± 367*	*1180 ± 76*	**2.0** ± 0.1	*650 ± 93*	**3.7** ± 0.5	*592 ± 153*	**4.1** ± 0.9
Nicotine	*3650 ± 1040*	*1380 ± 96*	**2.7** ± 0.2	*1390 ± 117*	**2.6** ± 0.2	*557 ± 226*	**7.5** ± 3.5

Data represent average ± standard deviation (s.d.) values of three independent experiments with each experiment comprising n = 4 replicates per data point.

## Discussion

Our study shows that the concept of HT mutagenesis can be applied not only to monomeric proteins [1,2], but also to complex multimeric receptors such as the muscle nAChR. The feasibility of a mutational screen depends on the feasibility of a functional assay based on transient transfection of monomeric genes. For the pentameric muscle nAChR this initially presented a challenge, and novel multigene expression plasmids had to be designed in order to establish a screening assay, as more classical transfection and assay optimization approaches proved ineffective. The aim of our study was to show the feasibility of the mutagenesis approach for the α1 subunit, which is incorporated into the pentamer in two copies. Future studies can utilize the same concept in order to expand to the β, δ, and ε subunits for a more thorough understanding of structure-function relationships of the muscle nAChR.

The library of 2816 mutants contained mutants for each of the 457 amino acids of the human α1 subunit, and unique and total coverages determined by SMRT sequencing 3.4 and 7.9, respectively. Despite the high quality of the library, mutational bias cannot be avoided. Limitations arise because of the low probability of achieving two nucleotide changes within the same amino acid codon, and indeed only single nucleotide changes per codon were detected in our study. Furthermore, the extent of codon degeneracy is different for each amino acid. For some amino acid there is no degeneracy, as they have only one codon (methionine and tryptophan). A single nucleotide change in this case must result in an amino acid change. Other nucleotide changes are much more likely to remain silent (*e*.*g*. arginine, serine and leucine which have six codons). 139 out of 143 possible residue changes that could be produced by a single nucleotide mutation were found to be present in the mutant library. The four missing changes are isoleucine to arginine, serine to tryptophan, arginine to methionine, and arginine to threonine, which are all unlikely events due to codon restrictions. Altogether, this demonstrates the importance of minimizing bias at the nucleotide level, given that the introduction of further systematic bias is unavoidable at the amino acid level.

The functional α1 subunit mutant screen was aimed at finding residues interacting with the two orthosteric blockers α-BTX and/or tubocurarine. However, none of the mutant hits (D44/L278, Y233, I235, V275) are in the known orthosteric binding site, and thus they do not overlap with the residues that are known to interact with these two blockers [7–9]. The SMRT sequence analysis showed that the mutant library had a broad coverage in this area, and included 130 mutants (24 of which are single amino acid mutants) of the 15 amino acids reported to interact with α-BTX [9]. Thus lack of coverage cannot explain why these positions have not been identified. One explanation could be species-related pharmacological differences. For instance, at the best characterised α-BTX binding position T209 mouse receptors have a phenylalanine and are very sensitive to α-BTX, whereas human receptors have a threonine and are less sensitive, and mongoose and cobra receptors have an asparagine and are completely insensitive [7]. Hence, the type of amino acid introduced by mutation (for the human α1 mutant library the only single amino acid mutant was T209S) can impact α-BTX interaction. For tubocurarine, the greatest mutation effect reported in the literature for an α1 residue is seen with the Y218T mutation, which decreases binding affinity at the high affinity site by 175-fold [8]. The mutant library did not contain any single point mutant for Y218. Interestingly, other types of mutation at Y218 are described to increase tubocurarine affinity [19], indicating that a broad spectrum of amino acid changes is desirable for each residue. In the present study, 40% of tested mutants did not show any detectable agonist response. This could be because the mutants may have not folded properly, and were not displayed at the cell surface. This is a common situation in many membrane proteins including ion channels, for which naturally occurring single amino acid mutants are defective in folding and plasma membrane trafficking, and thus cause human diseases [20,21]. Another possibility, besides misfolding, is that these mutants have lost the capability to respond to epibatidine. By definition, competitive antagonists interact with the same site as the agonist. Work on the α and γ subunits of *Torpedo* AChR suggested substantial overlap between residues important for tubocurarine binding and those important for agonist binding [19]. In addition to that, it is known that the complementary subunits are major determinants of tubocurarine affinity [4], and it could be that most of the single mutations in the α1 subunit have only minor effects and would be detectable only if associated with other mutations of interacting residues in the α1 subunit, and/or in the δ/ε subunits.

Somewhat unexpectedly, the specific mutations identified by the screen were found to have a GoF mode of action. For all three single amino acid mutants tested, we saw a significant enhancement in the potencies of a panel of muscle nAChR agonists. As a result of the potency shifts, the agonist concentration used in the screen to detect antagonist blockade became a saturating or supra-saturating concentration at the mutant channels. It is likely that this increase in the effective concentration of the agonist gave rise to the apparent resistance to the antagonists. Mutations at position V275 have been extensively described in the muscle nAChR literature and shown to have a GoF phenotype, giving rise to spontaneous openings. Mutations in this position in the related α7 nicotinic channel also give rise to a GoF phenotype [22,23]. This valine (13’ in transmembrane domain 2 (TM2)) is part of the hydrophobic girdle that starts at leucine 9’. Mutations in the equivalent position in the human β1 (V266M, V266A) as well as the ε subunit (V265A) give rise to a slow channel form of congenital myasthenia, displaying slower closing rates, spontaneous openings and an increase in apparent acetylcholine affinity [24,25]. Recently published further analysis of such β1 and ε subunit mutants revealed an increase in gating efficiency, due to increased opening rate and decreased closing rate. This study found the agonist affinity of mutant channels unchanged [26]. Our hits included another TM2 mutation, L278Q (position 16’ in TM2) that is likely to be a GoF mutation [27], even though this particular mutant had a second mutation (D44N). The two remaining GoF mutants identified are both located in the first TM domain of the α1 subunit (Y233N/H and I235M; 3’ and 5’ in TM1 respectively). To our knowledge, the effect of mutations at these positions has not been previously described in the extensive literature on muscle nAChR, demonstrating the value of our random mutagenesis approach. Ca^2+^ flux data do not allow a detailed mechanistic analysis for a fast channel like the muscle nAChR, largely because of the slow speed of agonist application by injection into the well and the limited time resolution. Nevertheless, the greater potency increase for the more potent agonists is precisely what one would expect, if the mutations acted by facilitating receptor gating. This pattern is predicted using receptor occupancy models with minimal assumptions [28].

Beyond the first HT mutagenesis for a multimeric protein, the study also shows the strength of detailed individual mutant sequence information obtained by SMRT sequencing. Not only the type of residue mutations covered is accurately determined, but also the exact combinations within each mutant. This corroborates the mutant interpretation, by helping us to identify the most interesting mutations in hits containing more than one residue alteration. In the study here, SMRT sequencing was performed based on four pools of the α1 subunit mutants following the functional mutant screen. Future mutagenesis studies could be advanced using bar-coded PCR primers for each mutant. SMRT sequencing with barcoded samples has been recently described [29,30]. This would enable to associate the SMRT sequence reads to each individual mutant located in a multi-well plate, and therefore to directly link sequence information with the functional signals. With such a set-up one could easily re-analyze potential false-negative mutant hits in the assay without having to retest the whole mutant library. Another possibility could be site-directed mutagenesis of each amino acid, which became recently more affordable and allows generation of a library containing only single amino acid mutants. In this context, one frequently sees alanine scans being performed, *i*.*e*. each residue is mutated into alanine, in the hope that this most likely maintains the protein conformation [31,32]. On the other hand, advantages and disadvantages of site-directed residue scans versus random mutagenesis remain to be substantiated by further investigations.

## Supporting Information

S1 Fig Establishment of a Ca^2+^ flux assay for transiently expressed muscle nAChRs. (A) Schematic illustration of the cDNA plasmids used to transiently express the four wildtype muscle nAChR subunits. (B) HEK293 cells were transfected with either a β1δε triple subunit plasmid, four single subunit plasmids (2:1:1:1 ratio), the quadruple α1β1δε subunit plasmid, or a β1δε triple subunit plasmid with a single α1 subunit plasmid (1:1 ratio). 48 h after transfection, cells were challenged with 6 μM epibatidine, and Ca^2+^ responses derived. Each bar represents the average (± s.d.) of four assay wells. (C) Typical epibatidine-stimulated concentration response curve obtained from HEK293 cells transiently transfected with the β1δε triple and single α1 subunit plasmids (n = 5, ± s.d.) using the Ca^2+^ flux assay. EC_50_ and EC_80_ concentrations are indicated. (D) Inhibitory concentration response curves of α-BTX and tubocurarine (n = 4, ± s.d.) obtained with Ca^2+^ flux measurements. Inhibitors were added 30 min prior to addition of epibatidine (EC_80_ concentration).
(TIF)

S2 Fig Sequence alignment of human and mouse muscle nAChR α1 proteins. Indicated in green are the β-sheets (β1-β10), in black the three ligand-binding loops (A-C), and in brown transmembrane domains (TM)1-4. Blue arrows show the 15 mouse α1 residues described to directly interact with α-BTX, and red arrows indicate the three residues that beared GoF mutations (Y233, I235, V275) in the present study.
(TIF)

S3 Fig Amino acid and nucleotide mutations around α1-V275 based on SMRT sequencing. (A) Number of mutants in the α1 subunit library within a corresponding 21-nucleotide stretch around residue 275. Colors indicate the type of nucleotide introduced by mutation. (B) Number of amino acid mutants in the mutant library within a 19 residue stretch around valine 275. Colors indicate the type of amino acid introduced by mutational change, and (*) indicates a stop codon.
(TIF)

S1 Table All six α1-Y233 mutants present in the α1 subunit mutant library. Mutant-3, mutant-4, and mutant-8 were discovered in the toxin screen, whereas the other three α1-Y233 mutants present in the library based on SMRT sequencing were not found. Nucleotide alterations are indicated in italics, and silent amino acid changes in grey. The number (n°) of SMRT reads reflects the confidence of sequence determinations. (*) indicates a stop codon.
(DOCX)

S2 Table All 11 α1-I235 mutants present in the α1 subunit mutant library. Mutant-7 was discovered in the toxin screen, whereas the other ten α1-I235 mutants present in the library based on SMRT sequencing were not detected. Nucleotide alterations are indicated in italics, and silent amino acid changes in grey. The number (n°) of SMRT reads reflects the confidence of sequence determinations. (*) indicates a stop codon.
(DOCX)

S3 Table All 11 α1-D44/L278 mutants present in the α1 subunit mutant library. Mutant-2 was discovered in the toxin screen, whereas the other seven α1-D44 mutants and three α1- L278 mutants present in the library based on SMRT sequencing were not detected. Nucleotide alterations are indicated in italics, and silent amino acid changes in grey. The number (n°) of SMRT reads reflects the confidence of sequence determinations. (*) indicates a stop codon.
(DOCX)
