# Supplementary figures and images for: High Throughput Random Mutagenesis and Single Molecule Real Time Sequencing of the Muscle Nicotinic Acetylcholine Receptor
Source: PLoS One. 2016 Sep 20;11(9):e0163129. doi: 10.1371/journal.pone.0163129 (PMC5029940; doi:10.1371/journal.pone.0163129)

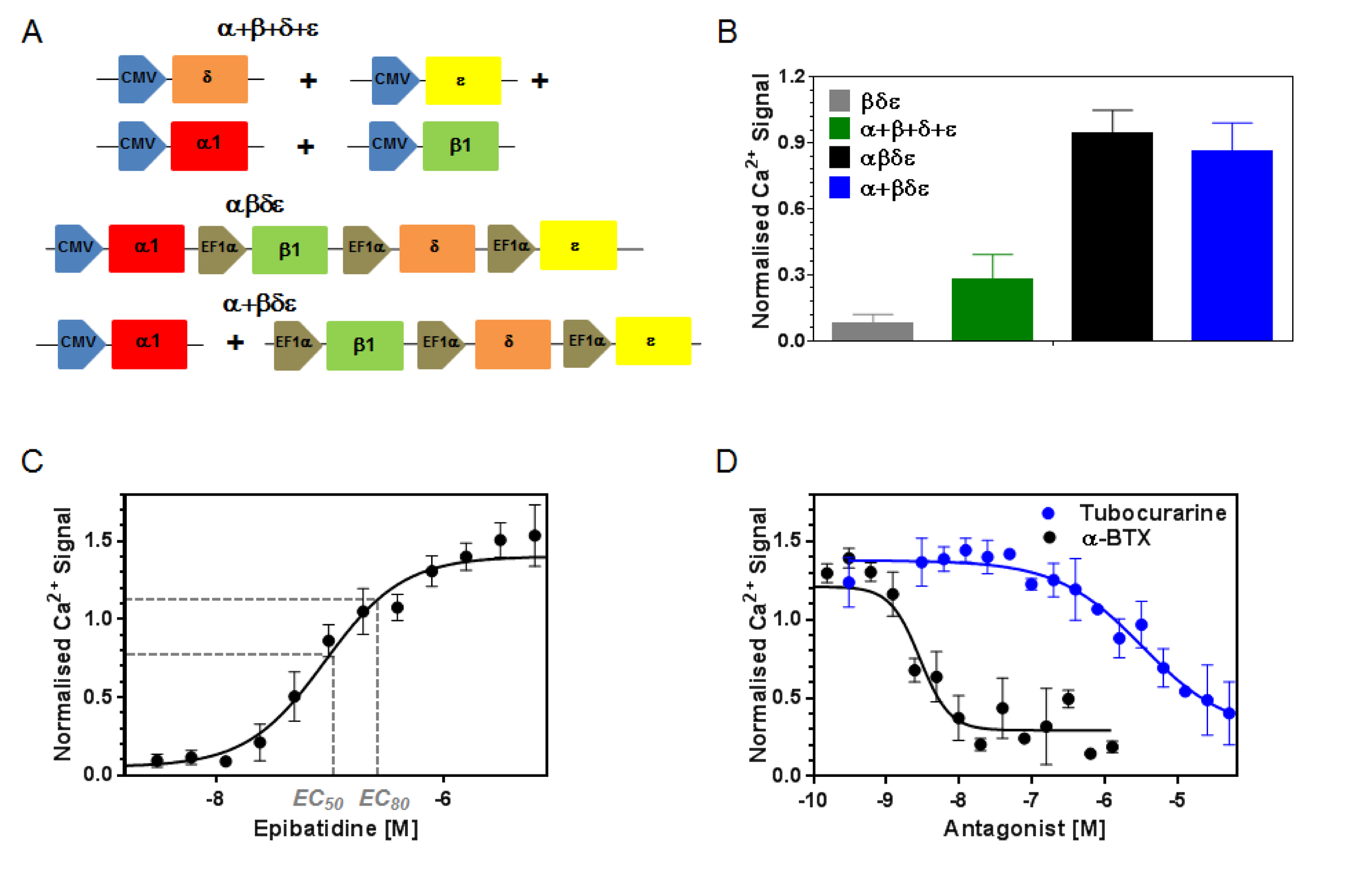

Supplement: S1 Fig — (A) Schematic illustration of the cDNA plasmids used to transiently express the four wildtype muscle nAChR subunits. (B) HEK293 cells were transfected with either a β1δε triple subunit plasmid, four single subunit plasmids (2:1:1:1 ratio), the quadruple α1β1δε subunit plasmid, or a β1δε triple subunit plasmid with a single α1 subunit plasmid (1:1 ratio). 48 h after transfection, cells were challenged with 6 μM epibatidine, and Ca2+ responses derived. Each bar represents the average (± s.d.) of four assay wells. (C) Typical epibatidine-stimulated concentration response curve obtained from HEK293 cells transiently transfected with the β1δε triple and single α1 subunit plasmids (n = 5, ± s.d.) using the Ca2+ flux assay. EC50 and EC80 concentrations are indicated. (D) Inhibitory concentration response curves of α-BTX and tubocurarine (n = 4, ± s.d.) obtained with Ca2+ flux measurements. Inhibitors were added 30 min prior to addition of epibatidine (EC80 concentration). (TIF) [file pone.0163129.s001.tif]

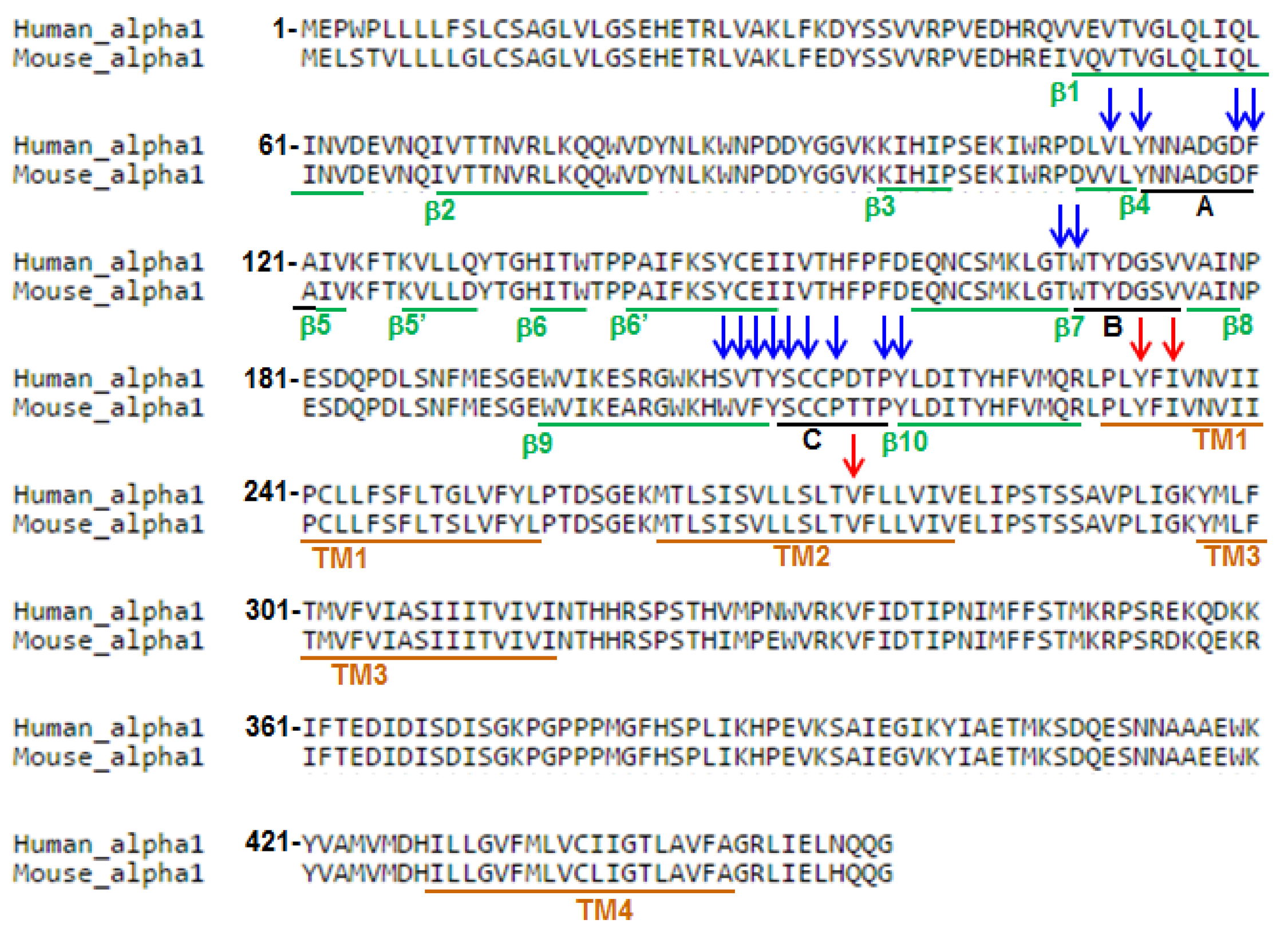

Supplement: S2 Fig — Indicated in green are the β-sheets (β1-β10), in black the three ligand-binding loops (A-C), and in brown transmembrane domains (TM)1-4. Blue arrows show the 15 mouse α1 residues described to directly interact with α-BTX, and red arrows indicate the three residues that beared GoF mutations (Y233, I235, V275) in the present study. (TIF) [file pone.0163129.s002.tif]

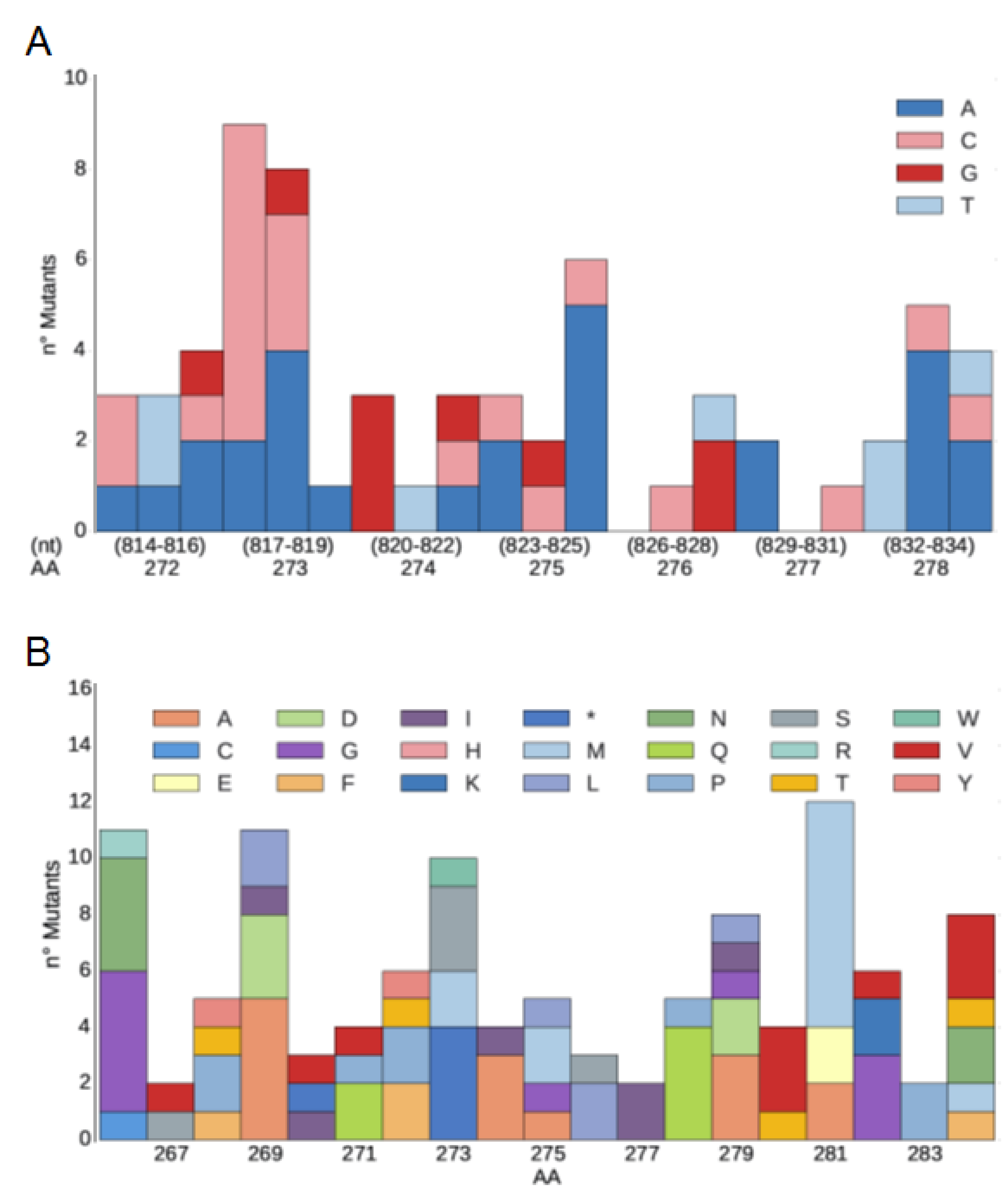

Supplement: S3 Fig — (A) Number of mutants in the α1 subunit library within a corresponding 21-nucleotide stretch around residue 275. Colors indicate the type of nucleotide introduced by mutation. (B) Number of amino acid mutants in the mutant library within a 19 residue stretch around valine 275. Colors indicate the type of amino acid introduced by mutational change, and (*) indicates a stop codon. (TIF) [file pone.0163129.s003.tif]
