# Supplementary material for: High Throughput Random Mutagenesis and Single Molecule Real Time Sequencing of the Muscle Nicotinic Acetylcholine Receptor
Source: PLoS One. 2016 Sep 20;11(9):e0163129. doi: 10.1371/journal.pone.0163129 (PMC5029940; doi:10.1371/journal.pone.0163129)
Supplement: S1 Table — Mutant-3, mutant-4, and mutant-8 were discovered in the toxin screen, whereas the other three α1-Y233 mutants present in the library based on SMRT sequencing were not found. Nucleotide alterations are indicated in italics, and silent amino acid changes in grey. The number (n°) of SMRT reads reflects the confidence of sequence determinations. (*) indicates a stop codon. (DOCX) [file pone.0163129.s004.docx]

| **Mutant** | **Mutated AA** *(nt)* | | | | | ***n°* Reads** |
| --- | --- | --- | --- | --- | --- | --- |
| **Mutant-3** |  |  | **Y233H** *(T697C)* |  |  | 14 |
| **Mutant-4** |  | silent *(C603A)* | **Y233H** *(T697C)* |  |  | 14 |
| **Mutant-8** |  |  | **Y233N** *(T697A)* |  |  | 24 |
| SMRT-20 |  |  | **Y233H** *(T697C)* | silent *(T1083A)* |  | 7 |
| SMRT-21 | **Y147C** *(A440G)* | **A177S** *(G529T)* | **Y233N** *(T697A)* |  |  | 8 |
| SMRT-22 |  | **I198T** *(T593C)* | **Y233*** *(C699A)* | **L248S** *(T743C)* | **T287S** *(A859T)* | 29 |
