# Supplementary material for: High Throughput Random Mutagenesis and Single Molecule Real Time Sequencing of the Muscle Nicotinic Acetylcholine Receptor
Source: PLoS One. 2016 Sep 20;11(9):e0163129. doi: 10.1371/journal.pone.0163129 (PMC5029940; doi:10.1371/journal.pone.0163129)
Supplement: S2 Table — Mutant-7 was discovered in the toxin screen, whereas the other ten α1-I235 mutants present in the library based on SMRT sequencing were not detected. Nucleotide alterations are indicated in italics, and silent amino acid changes in grey. The number (n°) of SMRT reads reflects the confidence of sequence determinations. (*) indicates a stop codon. (DOCX) [file pone.0163129.s005.docx]

| **Mutant** | **Mutated AA** *(nt)* | | | | | | | | ***n°* Reads** |
| --- | --- | --- | --- | --- | --- | --- | --- | --- | --- |
| **Mutant-7** |  |  | **I235M** *(C705G)* |  |  |  |  |  | 11 |
| SMRT-23 | **V49E** *(T146A)* | silent *(C687T)* | **I235N** *(T704A)* | **K400N** *(G1200C)* | **C438Y** *(G1313A)* |  |  |  | 12 |
| SMRT-44 |  |  | **I235V** *(A703G)* |  |  |  |  |  | 8 |
| SMRT-25 |  |  | **I235V** *(A703G)* | **I284V** *(A850G)* | **V291M** *(G871A)* | **V303G** *(T908G)* | **K400N** *(G1200C)* | silent *(T1344A)* | 20 |
| SMRT-26 |  | **E200G** *(A599G)* | **I235T** *(T704C)* | **P352S** *(C1054T)* |  |  |  |  | 14 |
| SMRT-27 |  |  | **I235T** *(T704C)* |  |  |  |  |  | 16 |
| SMRT-28 |  |  | **I235T** *(T704C)* |  |  |  |  |  | 13 |
| SMRT-29 |  |  | **I235F** *(A703T)* | **S268Y** *(C803A)* | **R449*** *(C1345T)* |  |  |  | 17 |
| SMRT-30 |  |  | **I235F** *(A703T)* | silent *(G729A)* | silent *(C811T)* | **M302V** *(A904G)* |  |  | 10 |
| SMRT-31 |  |  | **I235F** *(A703T)* |  |  |  |  |  | 19 |
| SMRT-32 |  | silent *(G147C)* | **I235F** *(A703T)* |  |  |  |  |  | 18 |
