# Supplementary material for: High Throughput Random Mutagenesis and Single Molecule Real Time Sequencing of the Muscle Nicotinic Acetylcholine Receptor
Source: PLoS One. 2016 Sep 20;11(9):e0163129. doi: 10.1371/journal.pone.0163129 (PMC5029940; doi:10.1371/journal.pone.0163129)
Supplement: S3 Table — Mutant-2 was discovered in the toxin screen, whereas the other seven α1-D44 mutants and three α1- L278 mutants present in the library based on SMRT sequencing were not detected. Nucleotide alterations are indicated in italics, and silent amino acid changes in grey. The number (n°) of SMRT reads reflects the confidence of sequence determinations. (*) indicates a stop codon. (DOCX) [file pone.0163129.s006.docx]

| **Mutant** | **Mutated AA** *(nt)* | | | | | | | | ***n°* Reads** |
| --- | --- | --- | --- | --- | --- | --- | --- | --- | --- |
| **Mutant-2** |  | **D44N** *(G130A)* |  |  | **silent** *(C402T)* | **L278Q** *(T833A)* |  |  | 13 |
| SMRT-33 |  | **D44N** *(G130A)* |  |  | silent *(G177A)* |  | **L293M** *(T877A)* | **T348K** *(C1043A)* | 14 |
| SMRT-34 |  | **D44G** *(A131G)* |  |  | **F247S** *(T740C)* |  | silent *(A1023G)* |  | 10 |
| SMRT-35 | **L6I** *(C16A)* | **D44G** *(A131G)* |  |  | **Q78R** *(A233G)* |  | silent *(A1119C)* | silent *(A1371G)* | 11 |
| SMRT-36 |  | **D44G** *(A131G)* |  |  | **Y132C** *(G393A)* |  | **N317Y** *(A395G)* | silent *(A949T)* | 11 |
| SMRT-37 |  | **D44G** *(A131G)* |  |  |  |  |  |  | 7 |
| SMRT-38 |  | **D44Y** *(G130T)* |  |  |  |  | **I402F** *(A1204T)* |  | 28 |
| SMRT-39 |  | **D44Y** *(G130T)* |  |  | **E103*** *(G307T)* |  |  |  | 4 |
| SMRT-40 |  |  | **A142D** *(C425A)* |  | **S174Y** *(C521A)* | **L278Q** *(T833A)* | **K360M** *(A1079T)* |  | 9 |
| SMRT-41 | **K30E** *(A88G)* | silent *(G372A)* | **C148*** *(T444A)* | **G194E** *(G581A)* | **L265R** *(T794G)* | **L278Q** *(T833A)* |  |  | 14 |
| SMRT-42 | **W4C** *(G12T)* |  | **C148W** *(T444G)* |  | **R229C** *(C685T)* | **L278P** *(T833C)* | **M328T** *(T983C)* |  | 13 |
